# Commentary: Adverse Childhood Experiences and Risk for Suicidal Behavior in Male Iraq and Afghanistan Veterans Seeking PTSD Treatment

**DOI:** 10.3389/fpubh.2017.00072

**Published:** 2017-04-10

**Authors:** Leo Sher

**Affiliations:** ^1^James J. Peters Veterans’ Administration Medical Center, Bronx, NY, USA; ^2^Icahn School of Medicine at Mount Sinai, New York, NY, USA

**Keywords:** adverse childhood experience, veteran, posttraumatic stress disorder, suicidal behavior, men’s mental health

The authors of a recently published article entitled, “Adverse childhood experiences and risk for suicidal behavior in male Iraq and Afghanistan veterans seeking PTSD treatment” by Carroll et al. (1) examined a range of adverse childhood experiences (ACEs) among Iraq/Afghanistan veterans with combat-related posttraumatic stress disorder (PTSD). The researchers found that the majority of veterans had experienced multiple types of adversities during childhood and/or adolescence. More than 80% of veterans reported experiencing at least one childhood trauma or adversity. About 40% of study participants endorsed four or more childhood traumas or adversities. Veterans who reported physical neglect as a child were significantly more likely to report a history of attempting suicide. It is important to note that studies of civilian populations suggest that ACEs are very strong correlates of adulthood suicide risk (2–4).

Research on psychiatric disorders and suicide risk among active duty personnel and military veterans tends to focus on traumas related to the military service (5–7). However, a focus on service-related traumas does not provide a complete picture. The observations by Carroll et al. (1) are consistent with reports that pre-military experiences such as adversity in childhood play an important role in post-deployment mental health (7–9). For example, Van Voorhees et al. (9) studied about 1,300 veterans and active duty soldiers to examine the link of childhood abuse with adult PTSD after taking into account combat experience. Forty percent of the sample reported at least one childhood traumatic event. The authors observed that ACE and adult combat experience independently influenced PTSD symptomatology.

The results of studies by Blosnich et al. (10) and Afifi et al. (11) indicate that the military service may be a way for some people to replace dysfunctional home environments with more structured and directive environments. It is worth noting that there may be other reasons why people with a history of ACE may seek a military environment later in life. Blosnich et al. (10) compared the prevalence of childhood adversities among persons with and without a history of military service in the United States. They used the ACE inventory to assess 11 negative experiences before the age of 18 years. In the all-volunteer era (since 1973), men with a history of military service had a higher prevalence of ACEs in all 11 categories than men who did not serve in the military. A similar observation was made by Afifi et al. (11) who compared child abuse exposure in Canadian Armed Forces personnel and in the Canadian general population. They found that “individuals with a child abuse history may be more likely to enter the military, and child abuse exposure may increase the likelihood of suicide-related outcomes.” It has been proposed that military enlistment among survivors of childhood adversities may be a sign of resilience and that the structure, training, and camaraderie of the military strengthen resilience among some survivors of childhood adversities (7). It is important to note that a study by Woodruff et al. (12) showed that most people who enlist in the United States Military do so for positive motives including patriotism, altruism, and self-improvement.

There are a lot of research observations of the effects of ACE on the psychobiological development of children and adolescents. Considerable evidence suggests that the brain development of children is likely to be affected in by their ACE (13–21). Early stress leads to an ongoing dysregulation of the hypothalamic–pituitary–adrenal axis stress response system (16–19, 22). It has been proposed that the stress response system can either become chronically overactivated or underresponsive (16–18, 22, 23). Neuropsychological studies frequently show that children who have experienced ACE have cognitive problems in one or more areas, when compared to children who haven’t experienced these adversities (14, 17, 18, 24). Many children that experience adversity develop difficulties related to learning, memory, and attention (13, 24–27). There is also evidence that social and emotional information is dealt with in a different way among persons with a history of ACE in comparison to individuals without a history of ACE (16–18, 28). Executive function difficulties can also develop as a result of ACE (18, 29, 30). The psychobiological development of individuals with ACE may determine their future life choices including the decision whether or not to join the military.

Military service may subject individuals with a history of trauma to additional trauma such as combat, which may additively increase risks of psychiatric disorders and suicidal behavior. If some military recruits have trauma-related psychological abnormalities that predispose to psychiatric disorders when they enter the military, then we have to seriously question the screening that goes into allowing persons to enter the military (31). Possibly, some individuals who were significantly traumatized by ACE and have psychiatric symptoms should be given non-combat assignments.

It is important to address early trauma histories in addition to more recent traumatic experiences because the psychobiological impact of early trauma is different from psychobiological effects of trauma during adulthood (32, 33). Studies have shown that a history of ACE is associated with poor response to treatment in patients with psychiatric disorders (34–36). Mental and non-mental health professionals who treat post-deployed service members should not only assess the seriousness of combat exposure but also childhood adversities to provide appropriate psychiatric treatment and to better understand psychiatric outcomes. Management of individuals with a history of ACE should include psychotherapeutic and pharmacological treatment of psychiatric disorders (37, 38). The aims of psychotherapy should include handling the disclosure of ACE in an open and empathic way, enhancing the patient’s sense of safety, helping patients to decrease current life stress, and improve their social support.

Clinicians, researchers, and health policy makers should work to reduce the stigma of revealing ACE and apportion funds for epidemiologic studies and the development of new treatment modalities to tackle problems related to ACE among military personnel and veterans (7).

## Author Contributions

The author confirms being the sole contributor of this work and approved it for publication.

## Conflict of Interest Statement

The author declares that the research was conducted in the absence of any commercial or financial relationships that could be construed as a potential conflict of interest.
